# Substandard and Falsified Medicines: Proposed Methods for Case Finding and Sentinel Surveillance

**DOI:** 10.2196/29309

**Published:** 2021-08-16

**Authors:** Elizabeth Pisani, Amalia Hasnida, Mawaddati Rahmi, Maarten Olivier Kok, Steven Harsono, Yusi Anggriani

**Affiliations:** 1 Erasmus School of Health Policy and Management Erasmus University Rotterdam Netherlands; 2 School of Public Health Imperial College London United Kingdom; 3 Faculty of Pharmacy Universitas Pancasila Jakarta Indonesia; 4 Department of Health Sciences Vrije Universiteit Amsterdam Amsterdam Netherlands; 5 IQVIA Public Health Singapore Singapore

**Keywords:** substandard drugs, falsified medicine, counterfeit medicine, medicine quality, sentinel surveillance, public health surveillance, substandard, pharmaceuticals, surveillance, public health

## Abstract

The World Health Organization and others warn that substandard and falsified medicines harm health and waste money, especially in low- and middle-income countries. However, no country has measured the market-wide extent of the problem, and no standardized methods exist to estimate the prevalence of either substandard or falsified medicines. This is, in part, because the task seems overwhelming; medicine markets are huge and diverse, and testing medicines is expensive. Many countries do operate some form of postmarket surveillance of medicine, but their methods and goals differ. There is currently no clear guidance on which surveillance method is most appropriate to meet specific public health goals. In this viewpoint, we aimed to discuss the utility of both case finding and risk-based sentinel surveillance for substandard and falsified medicines, linking each to specific public health goals. We posit that choosing the system most appropriate to the goal, as well as implementing it with a clear understanding of the factors driving the production and sale of substandard and falsified medicines, will allow for surveillance resources to be concentrated most efficiently. We adapted principles used for disease outbreak responses to suggest a case-finding system that uses secondary data to flag poor-quality medicines, proposing risk-based indicators that differ for substandard and falsified medicines. This system potentially offers a cost-effective way of identifying “cases” for market withdrawal, enhanced oversight, or another immediate response. We further proposed a risk-based sentinel surveillance system that concentrates resources on measuring the prevalence of substandard and falsified medicines in the risk clusters where they are most likely to be found. The sentinel surveillance system provides base data for a transparent, spreadsheet-based model for estimating the national prevalence of substandard and falsified medicines. The methods we proposed are based on ongoing work in Indonesia, a large and diverse middle-income country currently aiming to achieve universal health coverage. Both the case finding and the sentinel surveillance system are designed to be adaptable to other resource-constrained settings.

## Introduction

### Background on Substandard and Falsified Medicines

In late 2017, World Health Organization’s (WHO’s) press department issued a press release with the bold headline: “1 in 10 medical products in developing countries is substandard or falsified” [1]. More recently, with governments scrambling to secure supplies of diagnostic tests, medicines, and vaccines to cope with the COVID-19 pandemic, WHO and others have issued new warnings stating that the world may face an increased threat of poor-quality medicines [2-5]. These include substandard medicines, which are made by registered pharmaceutical companies in regulated factories but do not meet the quality standards set out in their market authorization paperwork, either because they were poorly made or because they have degraded since manufacture. This increased threat of poor-quality medicines also includes falsified medicines, which are made, repackaged, or sold by criminals who seek deliberately to misrepresent the identity, composition, or source of the product [6].

Poor-quality medicines can use up family and national budgets without curing patients; indeed, they sometimes poison or kill people instead of curing them. Underdosing infectious pathogens also allows drug-resistant infections to spread [7,8]. Thus, if these “medicines” are indeed common, they may substantially undermine physical and financial health. Estimates based on available data for particularly well-studied molecules provide an order of magnitude: poor-quality antimalarials were estimated to cost US $130 million per year in a single region of the Democratic Republic of Congo, US $141.5 million in Zambia, and US $830 million across Nigeria. In the latter country, substandard antimalarials are estimated to contribute to 12,300 deaths per year [9-11]. A 2015 study reported that, in 39 sub-Saharan African countries, there were 122,350 deaths attributable to poor-quality antimalarials among children under 5 years of age. However, the authors noted that “there is considerable uncertainty surrounding our results because of gaps in data on case fatality rates and prevalence of poor-quality antimalarials” [12]. Similarly, the meta-analysis that gave rise to WHO's press release, which said that 10% of medical products in developing countries are substandard or falsified, is careful to note the many limitations of that estimate. This meta-analysis was based on studies of uneven sizes and methods, conducted largely in low-income countries with limited domestic pharmaceutical industries, and heavily skewed toward antimalarials and a few other medicines that most interest global health agencies. Even within that constrained pool and looking only at studies that included sample sizes of 50 or more, reported prevalence of substandard or falsified medicines ranged from 0% to 91% [13]. Reviews have reported similar data constraints and findings [14-18]. For example, Ozawa and colleagues [18] found that studies reported a prevalence of substandard or falsified medicines between 0.8% and 89% in Africa and a prevalence of between 0.7% and 50% in Asia.

WHO actively maintains a case-reporting system for substandard and falsified medical products, including medicines, contraceptives, vaccines, and point-of-sale diagnostics. For brevity, we use the term medicines throughout this paper to cover all these medical products. Regular training provided to individuals designated as in-country focal points increases the use of the system, but, similar to all case-reporting systems, it provides no information on denominators (the number of products inspected or tested), so interpretation of trends and comparisons between countries is difficult.

It may be that the problem of medicine quality is understated because of a vicious cycle of limited systematic measurement leading to limited visibility and limited awareness of the problem that in turn restricts resources available for systematic measurement. Alternatively, the problem may be that WHO and researchers are cherry-picking data to overstate the problem, perhaps for reasons of self-interest, as Hodges and Garnett [19] suggest.

We do not know which of these dynamics holds true. There is, to our knowledge, no clear understanding of the prevalence of substandard or falsified medicines in any single country, let alone across all the “developing countries,” as suggested by the press release’s headline. No country has yet made systematic estimates of the prevalence of substandard or falsified medicines across all therapeutic categories in its medicine market, and no standardized methods for calculating such an estimation yet exist.

In this viewpoint, we aimed to briefly review different approaches to surveillance and estimation in public health, discuss their relevance in the context of medicine quality, and lay out ideas for 2 potentially cost-minimizing methods that may improve our ability to measure or reduce the prevalence of poor-quality medicines, especially, in low- and middle-income settings.

### Approaches to Surveillance

#### Overview of Surveillance Systems

We follow WHO, United States Centers for Disease Control and Prevention, the World Bank, and others in defining public health surveillance as the ongoing and systematic collection and use of data to inform policy, plan and evaluate interventions, and improve health outcomes [20,21]. Surveillance systems monitor the prevalence of infectious and noncommunicable diseases; of disability; and, increasingly, of the behavioral, social, corporate, and environmental causes of ill-health. In addition, surveillance systems have, in recent decades, expanded to include the systematic monitoring of health system factors such as service use, prescription practices, or access to medicine.

Surveillance can take many forms, each serving a slightly different purpose within the catch-all definition of “improving health outcomes.” However, most can be categorized into either “passive” or “active” surveillance. Passive surveillance involves reporting events such as disease diagnoses as they arise. An early example of passive sentinel surveillance in the United States was the weekly reporting of diseases by designated physicians, which began in Massachusetts in 1874 [22]. The informatics era has greatly expanded the potential for secondary data to be used to inform public health decision-making. Examples include the use of both retail data of over-the-counter medicine sales and data from internet searches to flag potential disease outbreaks, and the use of medical-claims data to track trends in noncommunicable diseases [23,24]. Active surveillance tends to be more resource intensive, usually involving purposive data collection—often the collection and screening of blood or other biological samples or, more recently, medical imaging.

Active surveillance systems systematically collect and test samples for the purpose of tracking ill-health or health-related risks. Some active surveillance, such as active sentinel surveillance, test a cross-section of a defined population to establish disease prevalence. Others, such as case finding, specifically, target individuals at highest risk of needing services. These 2 types of active surveillance have different purposes. Sentinel surveillance, similar to many other surveillance systems, such as those that track noncommunicable diseases, determinants of health, and health system factors, provides data intended to guide medium- or longer-term health program planning. Case-finding systems, frequently used in infectious disease outbreaks but also used for early detection of treatable noncommunicable conditions, provide data intended to inform immediate therapeutic or preventative action. These different goals, upon which this paper focuses in the context of medicine quality, affect the design and use of surveillance systems and data, as show in Table 1.

**Table 1:** Major types of surveillance systems in public health.

Purpose	Outbreak response	Health program planning
System design:	Case finding: identify infected individuals	Sentinel surveillance: track prevalence over time
Resulting action:	Isolate and treat	Adjust policies and programs
Key characteristic:	Specific: pinpoint individuals for rapid follow-up	Comparable: standardized methods allowing comparison over time
Cannot be used to:	Estimate prevalence; track trends over time	Respond at individual level

#### Case Finding

The control of outbreaks and epidemics of infectious diseases requires that chains of transmission be broken. In these circumstances, surveillance systems try to identify infected individuals, isolating and, if possible, treating them to interrupt transmission. We term these systems “case finding.” They are relatively rare but have seen a resurgence during the COVID-19 pandemic.

#### Sentinel Surveillance

Sentinel surveillance systems are more common. Designed to track trends in infection over time, they use standardized methods to measure the prevalence of a disease within a defined population, comparing the result with prevalence measured in the same way in earlier years or in different locations. Sentinel surveillance is used to estimate the burden of disease, to target prevention and treatment interventions, and to monitor the impact of these interventions.

While passive surveillance can achieve these goals, it is of limited use for tracking rare diseases, which are easily missed by these systems [25]. While epidemiological orthodoxy holds that active surveillance involving regular screening of randomly selected samples provides the best approximation of disease trends across a population as a whole, this is also impractical for rare conditions that would require very large samples.

The HIV pandemic entrenched the idea of active sentinel surveillance in populations defined not by geography but by risk of exposure to the virus [26,27]. This allowed health authorities to focus surveillance resources in subpopulations where the majority of cases of the largely invisible disease were to be found while still producing comparable data and tracking trends over time. In many countries, those groups included people who inject drugs, gay men, sex workers of all genders, and sex workers’ most frequent clients.

**Figure 1:**
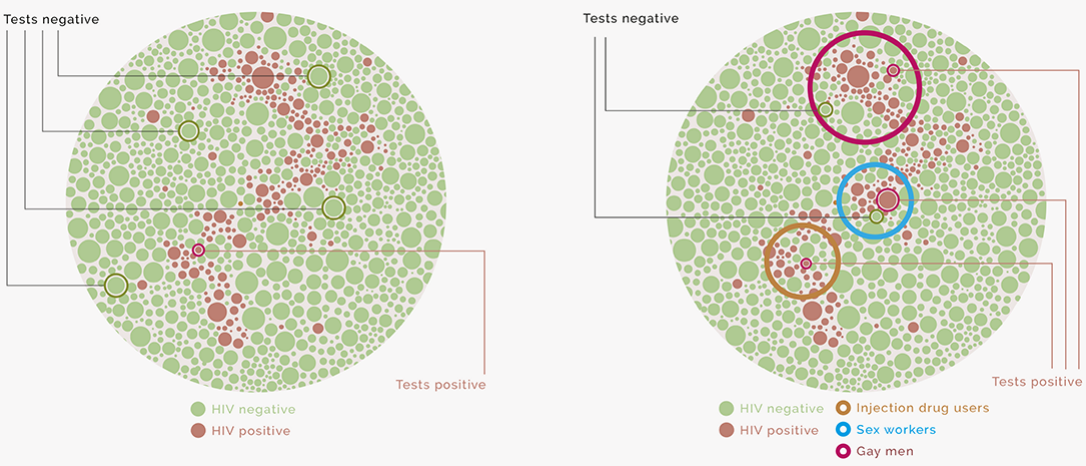
Illustrative difference between random and risk-based surveillance for HIV.

Figure 1 illustrates randomized and risk-based approaches to HIV sentinel surveillance. For the same limited resources (in this simplification, 5 tests), random sampling, on the left, yields just 1 positive test, while sentinel surveillance, on the right, yields 3. Combined with robust estimates of the size of those subpopulations, this approach will provide a more accurate estimate of the prevalence of infection nationally, the data produced will more accurately reflect the effect of targeted risk-reduction interventions, and these benefits will be achieved at a lower cost compared with random sampling.

The HIV example is of considerable relevance when thinking about surveillance of substandard and falsified medicines, because it is largely invisible until actively tested and clusters around known risk factors. A similar model for medicine quality is presented in this paper’s section “Proposed Method for Sentinel Surveillance.”

### Existing Surveillance of Medicine Quality

At the international level, surveillance of medicine quality takes the form of passive reporting of detected cases. As with disease case reporting, this provides information by demographic, geographic, environmental, or other factors, which is invaluable in helping to identify clusters of risk. However, case reporting does not provide any idea of the number of products tested. No case reports may mean there is no problem in a particular country, but it may also mean there is no capacity or willingness to detect or report cases.

At the national level, medicine regulators in many countries conduct some form of postmarket surveillance. In some countries, this is largely passive, limited to collating reports of adverse events submitted by health care providers through pharmacovigilance systems. Again, this means the denominator is unknown. However, in other countries, the regulator actively samples medicines from supply chains for inspection and testing. Where they report the number of products inspected or tested, as well as the number of out-of-specification products, this active surveillance allows for the calculation of prevalence in the segment of the market from which samples were drawn.

Sample selection in active surveillance varies widely, from random to convenience sampling, although regulators do not always state which method they use. Academic groups and WHO have published recommended methods for conducting surveys of medicine quality [28,29], as well as for sampling high-risk medicines from the internet [30]. While not focused specifically on sentinel surveillance, these methods have informed the guidance provided by technical and regulatory agencies on surveillance approaches that focus on selecting products at the highest risk for inspection and testing, including at the point of import [31-36]. To date, the criteria for determining risk have focused largely on the risk of impact to public health, factors intrinsic to the molecules (eg, stability and therapeutic index), and regulatory history. Not all agencies share information about risk profiling, for fear of helping those who produce poor-quality medicines to circumvent targets. However, as far as we know, market-related drivers of the risk of falsification are rarely considered. Furthermore, none of the guidelines or tools currently in the public domain explicitly differentiate between the risks for falsification and the risks for substandard production or degradation. However, attention paid to risk-based, postmarket surveillance is growing; the WHO Member State Mechanism on falsified and substandard medicines chose the development of methods and tools for risk-based surveillance as a prioritized activity in its current workplan [6], and work is ongoing.

Sentinel surveillance of substandard or falsified medicines is a form of postmarket surveillance designed explicitly to select samples in reproducible ways over time, so that trends can reliably be measured. This is rare in the case of substandard or falsified medicines, although some repeat random surveys have been conducted [37]. Site-based sentinel sampling has also been attempted in some locations. This may suffer from bias if people, including falsifiers, become aware of the practice and change behavior to avoid supplying known sentinel sites [29].

In the context of medicine quality, case-finding efforts focus on trying to identify individual products that are most likely to be substandard or falsified, so that they can quickly be recalled or otherwise removed from the market. In some high-income countries with strong pharmacovigilance systems, these efforts coexist with active sampling from the supply chain.

Market-wide estimates of the prevalence of substandard or falsified medicines are virtually nonexistent. Reasons for this include the apparent complexity of the task and the expense of pharmacopeial testing. Even small countries will typically have many thousands of registered medicines and vaccines on the market. Meanwhile, well-staffed medicine-testing laboratories are scarce—there are fewer than 50 WHO-prequalified drug-testing laboratories across all low- and middle-income settings [38]. Local pharmaceutical reference standards and reagents that allow for testing of the content and quality of medicines are often unavailable, while international, gold-standard products can cost several hundred dollars for even the most common molecules [39]. In addition, medicine regulators may be wary of systematic approaches, seeing transparent surveillance and robust estimation processes as an unwelcome evaluation of regulatory performance.

In short, while postmarket surveillance exists in different forms, there is currently no global guidance on the purpose or shape of national surveillance systems for substandard or falsified medicines and no standardized methods for translating the results of surveillance into market-wide estimates of prevalence.

The remainder of this paper proposes candidate methods, expanding on existing risk-based approaches. We aimed to review the risk factors that underlie (1) substandard and (2) falsified medicines; to propose a method for case finding based on the identified risk factors; to propose a sentinel surveillance method based on the identified risk factors; and to propose a method for developing nation-wide estimates for the prevalence of substandard and falsified medicines, based on sentinel surveillance. Our proposal is based on exploratory work undertaken in Indonesia. We believe the proposed methods are feasible in many resource-limited settings.

## Risk Factors for Poor-Quality Medicines

### Overview of Risk Factors for Poor-Quality Medicines

Both falsified and substandard medicines exist because there is money to be made selling them. In the same way that a spike in opportunistic infections once signalled a potential cluster of undetected HIV infections, dymanics in medicine markets can act as crude predictors of clustered cases of substandard or falsified medicines. In earlier works, we reviewed available academic literature; examined reports of the case-reporting database, WHO Global Surveillance and Monitoring System for substandard and falsified medical products; and conducted detailed case studies in 4 middle-income countries, using an epidemiological approach to identify risk factors associated with substandard and falsified medicines [6,40]. We identified a limited number of market-related factors that combine to increase the possibility that certain products in a market will be substandard, as shown in Table 2.

**Table 2:** Market risk factors for substandard medicines.

Risk factor	Risks to quality
High or rising pressure on profit margins	Incentivizes cost cutting
Stretched technical capacity	Increases risk of production errors or degradation during distribution
Limited oversight	Allows substandard products to flow through the supply chain
Low risk of damage to corporate reputation	Reduces incentive to invest in quality assurance

Some of these market factors operate at the level of a particular brand, others operate at the company level, others relate to the level of the supply chain, and others relate to a specific molecule. These market factors interact and further combine with other factors already considered in risk-based surveillance for medicine quality, such as the stability of a molecule or the complexity of the production or packaging process, to signal the likelihood that a medicine will be substandard or falsified.

A comprehensive review of academic literature describing interventions to control falsified medicines found few studies that addressed market drivers of falsification [41]. However, we find market-related factors strongly shape incentives for falsifiers, leading to increased risk of falsification, as shown in Table 3.

**Table 3:** Market risk factors for falsified medicines.

Risk factor	Falsifier incentive
Shortage of (or restricted access to) affordable, desired product	Criminals prefer to make and sell products where there is a ready market (where demand exceeds accessible supply)
High-priced medicine or relatively high-priced brand	Profit opportunity influences choice of product and brand falsification
Limited risk of discovery or punishment	Risk of retribution shapes choice of distribution channel

We propose adding indicators of the market factors shown in Tables 2 and 3 to increase the specificity of existing risk-based sampling and to more easily distinguish between products at risk for falsification and those more likely to be substandard.

### Proposed Method for Case Finding: Sample Based on an Index of Risk

Effective case-finding systems may appeal to regulators, politicians, and the public, because they inform product recalls and other immediate actions to protect patients. On the downside, these systems are data-hungry, and sampling is relatively resource intensive. They do not systematically test a specific number of samples from a well-defined population, and, thus, cannot easily be used to measure trends over time or to estimate the magnitude of the problem.

These limitations notwithstanding, many sources of routinely collected data related to medicine markets do exist, including in middle- and some lower-income countries. These include data collected by medicine regulators, health authorities and insurers, customs and excise departments, and market research firms. We propose to use these data to guide case finding, as shown in Textbox 1. Steps 2-5 should be undertaken separately for substandard and falsified medicines.

**Steps for systematic case finding for falsified and substandard medicines.**

- Step 1: Define indicators of public health importance (eg, burden of disease, vulnerability of affected population, narrow therapeutic index, sales volume of brand, or dosage form).
- Step 2: Define 1 or more objective indicators for each of the risk factors for substandard medicines and for falsified medicines, specifying the level at which it operates. Identify potential collinearity, and eliminate duplication.
- Step 3: Create risk scores for each numeric indicator (eg, none, minimal, some, or high), and calculate indicators and scores for each product (examples in Supplementary Tables A and B in [42]).
- Step 4: For each product, add risk scores to create a total index of risk. Select products to be sampled, prioritizing those with the highest risk index.
- Step 5: For sampled products, weigh by risks related to geography and supply chain, and draw up a sample frame.
- Step 6: Sample selected products (from specified locations, if indicated in Step 5).
- Step 7: Test sampled products. For potentially falsified products, screen visually and using rapid or low-cost devices such as a hand-held spectrometer or field-based thin layer chromatography. For potentially substandard products, perform quantitative assay and dissolution tests.

Step 1 is carried out in consultation with health authorities, while steps 2 and 3 take into account available data sources and the opinion of experts from the many sectors involved in the production, procurement, sale, and use of medicines. In Table 4, we provided a single example of a possible indicator for each area of risk for substandard production or degradation. These suggestions derive from ongoing exploratory work in Indonesia, a large middle-income country with substantial domestic medicine production and a single-payer health insurance system. Exact specifications of the indicators, as well as decisions about potential weighting, may differ by country and will be determined, in large part, by the data available. A more comprehensive list of alternative indicators, together with suggested data sources, is provided in the supplementary tables in [42].

Because the medicine market is extremely heterogeneous, several indicators use relative measures, such as ratios compared with the median. These indicators must then be turned into scores that can be added together to create a total index of risk as described in Textbox 1. Supplementary Table A in [42] suggests methods for turning indicators into risk scores.

To provide a single example for the first indicator in Table 4, examine the ratio of price to weighted market median price for the same product. If the ratio is above 1, the product is priced above the market median and, thus, not deemed irrationally cheap or at risk of cost cutting. Deciles of risk are calculated only for those products with a price–to–median-price ratio of less than one. Products closest to the median (deciles 7-10) may also be considered at no risk. Those in deciles 5 and 6 may score at 1 risk point (at minimal risk for cost cutting), and those in the second to fourth deciles score 2 points (at some risk). Brands (or nonbranded products from a specific market authorization holder) that fall into the first decile—the products selling at the deepest discount—are awarded 3 risk points (at high risk for cost cutting). Narrower gradations would allow for greater specificity; expert committees may decide what is most appropriate in the local context.

A similar process can be undertaken for products at risk for falsification, but the indicators will be different. Table 5 provides examples for each of the major risk-factor groupings. Again, a more comprehensive list of alternative indicators, together with suggested data sources, is provided in the supplementary tables in [42].

The success of the case-finding approach will depend, to a significant extent, on the willingness of data custodians to share these data with those conducting case finding. The sensitivity and specificity of case finding will additionally depend on the ways in which indicators are combined. While Textbox 1 describes a simple index, weighting is possible. If weighting is used, it is likely that brand-specific indicators, which have greater specificity, will carry a greater weight than market-wide indicators relating to molecules. However, we propose working with regulators to use retrospective data to find the model that best predicts poor-quality products in specific markets. Regulators with higher capacity for analysis may wish to develop more complex algorithms, including “big data” approaches, that combine price and volume data in ways that more closely pinpoint risk in specific markets.

**Table 4:** Indicative components to flag potentially substandard medicines.

Indicates	Indicator	Level at which indicator applies	Rationale
Profit pressure: cost cutting	Ratio of price to weighted market median for same product (same molecule and dosage form)	Brand and dosage form	Although premium brands are usually available, products produced by a large number of companies will tend toward the lowest cost of quality-assured production plus a fair profit [43]. If a particular product sells significantly below the market median, it may signal insufficient investment in quality assurance or other cost-cutting measures.
Technical limitation: production errors	Number of years continuously producing this molecule	Manufacturer (per molecule)	As companies and their staff gain experience and streamline their standard operating procedures in the production of a new medicine, the risk of production errors falls. Mistakes in production are more common among newly registered manufacturers.
Limited oversight	Time since most recent GMP^a^ inspection of any facility	Manufacturer (by production site)	Medicine regulators aim to inspect production facilities on a regular basis; some additionally include risk-based inspection. In practice, frequency of inspection depends on regulatory capacity, and intervals may vary. The risk of detectable deviations from GMP grows with time since last inspection.
Production history	Number of regulatory warnings or sanctions given to manufacturer over reference period	Manufacturer (all products)	Investment in quality assurance is embedded in corporate culture. Manufacturers who repeatedly receive warnings for GMP violations may systematically underinvest in quality assurance, meaning all their products are at higher risk.
Reputational risk	Number of years of MA^b^ holder in market	MA holder (all products)	Most companies are incentivized to invest in QA^c^, in part, because they wish to maintain their reputation as a provider of quality goods. New companies may be established opportunistically, especially, in rapidly growing markets. With less investment in building a reputation than older firms, new companies may have less to lose if found to be marketing substandard products.
Intrinsic risk: degradation	Stability of molecule	Molecule (all products)	Some molecules are less stable than others and more sensitive to variations in humidity, temperature, light, or other factors. Less stable molecules are more likely to degrade, becoming substandard before consumption.
Ecological risk: degradation	Classification of district accessibility	All products (or less stable products), by point of sale	Long supply chains and poor infrastructure pose challenges for maintaining temperature and humidity and may also reduce frequency of distribution. These factors increase the risk of degradation, especially, for less stable products.

^a^GMP: good manufacturing practice.

^b^MA: market authorization.

^c^QA: quality assurance.

**Table 5:** Indicative components to flag potentially falsified medicines.

Indicates	Indicator	Level at which indicator applies	Rationale
Market opportunity: limited affordability	Product is on patent but not listed in current national formulary	Brand	On-patent products usually have premium prices. When they are not listed in current national formularies, they are usually not covered in the national insurance scheme, indicating limited affordability for patients. Patients or health care providers may seek these products at cut prices outside of the regulated supply chain.
Market opportunity: desirability	Molecule is used recreationally or off-label	Molecule	Some narcotics and psychotropic medicines are used recreationally or otherwise abused, including use for purposes for which they are not licensed. Additionally, access to some medicines is tightly restricted for political reasons, such as their potential use as abortifacients. Since the sale of these products is regulated, users without prescriptions commonly seek them outside of the regulated supply chain or from vendors who do not observe due diligence.
Profitability	Ratio of (price × retail channel sales volume) to market median, for the same dosage form	Brand	Falsifiers want to sell products for which there is a lucrative market, for which a large number of patients are prepared to pay a high price. For any given medicine for which there is a choice of brands, those brands with a combination of a relatively high retail price and a relatively large sales volume will be attractive targets.
Low risk of detection	Number of listings for product on 2 largest internet marketplaces	Brand	General internet marketplaces provide an unregulated but commonly used space for trading medicines without official licenses. The vast number of online transactions creates difficulty for regulatory monitoring, and anonymity limits the possibility of repercussions. More listings of products on the largest general online marketplaces also indicate high demand.

### Proposed Method for Sentinel Surveillance: Tracking Trends in Risk Groups

Sentinel surveillance is a form of postmarket surveillance that is less data-intensive than case finding and has a different purpose. Systematic testing of comparable samples over time allows health authorities to: establish the likely prevalence of substandard medicines and, separately, of falsified medicines; inform estimates of the health and economic impacts of these medicines; make a case for additional investment in quality assurance in production or procurement, if necessary, including more investment in regulatory enforcement; plan and implement policies and programs to reduce prevalence of poor-quality medicines; and track progress over time toward achieving that goal.

The principal challenge in developing a robust, risk-based sentinel surveillance system for substandard and falsified medicines is in identifying “risk groups” of medicines, within which most poor-quality medicines cluster. These risk groups are the functional equivalent of the risk behaviors that circumscribe sentinel populations for another invisible threat to health, HIV infection.

Similar to HIV sentinel surveillance, the specific sentinel groups may vary from country to country, depending on market dynamics and the risks and opportunities they create. The critical point is that groups are defined based on the feasibility of drawing samples and in ways that are replicable over time.

Drawing from existing risk-based approaches and the additional market risk factors identified in Table 2 and Table 3, and, again, with reference to ongoing exploratory work in the Indonesian market, we propose sentinel groups for both substandard medicines (Table 6) and falsified medicines (Table 7). We underline that these groupings are not intended to encompass all at-risk products, nor do we suggest that all of the products in these groupings are at risk. Rather, these factors act as proxies that may yield a higher concentration of at-risk products, compared with a random sample.

**Table 6:** Suggested sentinel groups for substandard medicines.

Sentinel group	Definition	Signals potential problem of	Rationale
Irrationally low-priced essential medicines	In public systems, medicine price <75% of international reference price; in retail pharmacies, cheapest available version of target medicine	Cost cutting	Irrationally low prices are a strong predictor for cost cutting. Selection of samples from the public system can be brand specific, and price data are available in advance, so a clear price threshold can be set. For private provision and retail sampling, brand-specific sampling is not feasible, so the cheapest medicine available should be sampled. Molecules should be selected for local public health importance.
Contract- manufactured medicines	Products randomly selected from those that are manufactured by a company other than market authorization holder	Reduced oversight	Contract manufacturing is a frequent means of lowering production costs (by outsourcing to companies that can achieve economies of scale). The market authorization holder does not always have clear oversight of quality assurance practices at contract manufacturers.
Poor regulatory history	Medicines randomly selected from those made by companies with history of regulatory violations and involuntary recalls within a time reference period	Inadequate quality assurance	Though regulators work with past violators to improve manufacturing and distribution practice, corporate culture and incentives appear to act as enablers of cost cutting and other practices that increase the risk of substandard production or degradation.
Technically vulnerable	Medicines randomly selected from those that are technically challenging to manufacture or distribute, including those with unstable molecules, limited therapeutic index, or sterile forms	Higher potential for production errors	These product-specific characteristics require particular investment in quality assurance. It would be possible to restrict this sentinel group to newer, less experienced manufacturers or market authorization holders. For unstable molecules, samples may be drawn from outlets in geographically remote areas.

**Table 7:** Suggested sentinel groups for falsified medicines.

Sentinel group	Definition	Signals potential problem of	Rationale
High irrational demand	Medicines that are used for recreation or other off-label purposes, for which alternatives are restricted or expensive; randomly sampled from retail outlets	Market opportunity	Demand planning is based on authorized uses only. Off-label use creates shortages, which provide market opportunities for falsifiers. Sample frame may be weighted toward independent pharmacies and medicine shops.
Life-saving but unaffordable	Medicines that are known or reputed to be life-saving, that retail at >10% of average per capita household spending, but that are not covered by national insurers; brand-specific sample from retail outlets	Market opportunity	Patients with life-threatening conditions are highly motivated to acquire these medicines. High profit margins incentivize their sale, which may diminish due diligence even in the regulated supply chain. This sample may include products not locally authorized; these should also be screened for falsification.
Sold on unregulated internet platforms	Random sample of “prescription-only” medicines sold through unlicensed internet sellers (sentinel group may be combined with signals of profit potential though purposive sampling of brands retailing at >200% of market median for the dosage form)	Evasion of regulation	Falsifiers favor internet sales because the potential for detection and successful prosecution is low. While the sample may be weighted toward medicines with high irrational demand, it should include other medicines of public health importance, such as antibiotics.

Most sentinel groups should be sampled in the public, private, and (if applicable) nonprofit sectors; the obvious exceptions are samples specific to unregulated channels, which should not exist in the public sector. A sentinel surveillance system may be established following a process similar to that described in Textbox 2.

**Steps to establish sentinel surveillance for falsified and substandard medicines.**

- Step 1: Define indicators of public health importance (eg, burden of disease, vulnerability of population, sales volume of brand or dosage form, or publicly procured); list medicines by public health importance.
- Step 2: Review local data sources and market conditions to define proxy “sentinel groups,” in which the highest concentrations of (A) falsified and (B) substandard medicines of public health importance are likely to be found.
- Step 3: Define indicators (or combinations of indicators) that best circumscribe those sentinel groups.
- Step 4: Draw up a sample frame for each sentinel group, including in it the public and private sectors, as appropriate, and sample a predetermined number of products for testing.
- Step 5: Test samples. For sentinel groups containing potentially falsified products, screen visually and using rapid or low-cost devices such as hand-held spectrometers or field-based thin layer chromatography. For sentinel groups containing potentially substandard products, perform quantitative assays and dissolution tests.

**Figure 2:**
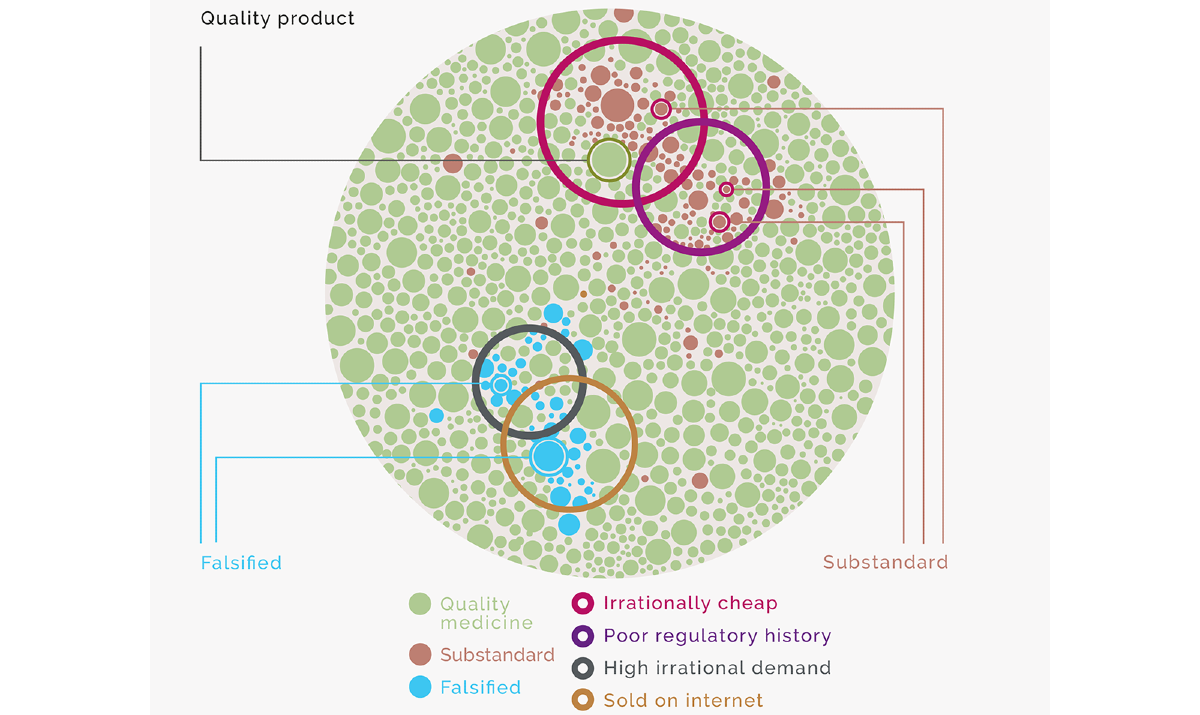
Illustration of risk-based sentinel surveillance for substandard and falsified medicines.

Pharmacopeial testing is expensive. The risk-based sentinel approach aims to reduce costs of routine surveillance in 2 ways. First, it increases the “yield” of testing by focusing it on the clusters of medicines most likely to be at risk (Figure 2). Second, it provides an initial triage for testing technologies. Products selected in sentinel groupings for falsification risk can be screened visually and using lower-cost field-based devices [44]; only those at high risk of substandard production need to undergo assay and dissolution testing.

### Developing National Estimates for the Prevalence of Substandard and Falsified Medicines

#### Steps to Estimate the Prevalence of Poor-Quality Medicines

In the same way that HIV prevalence among sex workers or drug injectors does not represent the prevalence of the virus in a whole population, the prevalence of poor-quality medicines in risk-based sentinel groups does not represent medicine quality across a whole national market. However, if the size of each of those sentinel groups can be calculated and if assumptions can be made about their relationships with the wider population, then sentinel surveillance can provide a starting point for making robust estimates of national prevalence [45]. The same is true for medicine surveillance groups and their relationships with the wider market. Textbox 3 suggests a process for developing such estimates.

**Steps to estimate the national prevalence of falsified and substandard medicines.**

- Step 1: Conduct risk-based sentinel surveillance for falsified and substandard medicines to obtain prevalence estimates for each sentinel group.
- Step 2: Calculate the interaction between sentinel groups.
- Step 3: Use market, procurement, and regulatory data to estimate the total volumes of medicines in each sentinel group.
- Step 4: Apply the prevalence estimates (step 1) to the volume data (step 3) to estimate the total number of substandard medicines across all sentinel groups for substandard medicines, correcting for interactions where necessary. Repeat for falsified medicines.
- Step 5: Use market, procurement, and regulatory data to estimate the total volumes of medicines in (lower-risk) market subsectors that do not fall in the sentinel groups.
- Step 6: Use all available data sources (eg, regulatory data, academic studies, case-reporting data, and data from other countries) to make assumptions about the residual prevalence in these nonsentinel sectors, comparing with prevalence in sentinel groups. Document each assumption, and, then, estimate the prevalence of substandard and falsified medicines in each sector.
- Step 7: Apply the prevalence estimates (step 6) to volumes (step 5) to estimate the total number of poor-quality medicines outside of sentinel groups.
- Step 8: Calculate the national prevalence estimate, where national prevalence estimate = (step 4 + step 6)/(step 3 + step 5).

The approach in Textbox 3 has the great advantage of transparency. In addition, the process will highlight important data gaps that can be rectified over time. Assumptions can easily be corrected as more complete data are collected or shared. For example, step 6 might initially include the assumption that imported, on-patent oncology drugs that do not fall into any of the groups in Table 6 are substandard 0% of the time. The assumption may change if regulators in producing countries issue product recalls for products that are also exported.

#### Estimation Process

Health-related estimates tend to improve in accuracy and local relevance (and, thus, potential utility) if potential end users are involved in their production [46]. This is, in part, because these actors can identify and contribute data to the process, and their expertise provides critical insights for informing necessary assumptions [47]. Estimation of the prevalence of substandard and falsified medicine should be led by medicine regulators and ministries of health. We would strongly encourage active consultation with pharmaceutical manufacturers and distributors, insurers, procurement authorities, consumer and patient advocates, and professional associations (eg, doctors and pharmacists) for deciding on methods and assumptions. The policies, decisions, and behaviors of these actors shape markets and influence the quality of medicines that patients consume [48]. Besides enriching the process, their participation increases the likelihood that estimates will be broadly accepted and acted upon. However, the motivations and interests of these groups are rarely aligned; careful annotation and transparent publication of all assumptions and data sources used in the estimation process can guard against capture by any interest group, protecting the integrity of the estimates [47].

#### Next Steps

The national estimates that result from this process will not capture the full complexity of medicine markets, especially, in initial rounds of estimation. However, we think it is important to begin to work toward that goal with tools that are most likely to be adopted by regulators in resource-constrained settings. We believe that a feasible and important first step in better quantifying the threat posed by substandard and falsified medicines includes simple, spreadsheet-based national models that clearly document all data sources and assumptions and that are based on clearly defined and repeatable sentinel surveillance. Later, more sophisticated models may embrace more complexity and be expanded to estimate the extent to which these products undermine health and well-being and the damage they do to family and national budgets.

Our suggested methods may seem complex, and the processes may seem institutionally daunting, but, again, we draw inspiration from the experience of HIV surveillance. The current state of information systems for medicine quality closely resembles HIV surveillance systems circa 1995. Many low- and middle-income countries had no system at all. In those that did, incomplete case reporting was the norm; sentinel surveillance focused mainly on pregnant women even in countries where most infections were in men; behavioral risk surveillance was in its infancy; and estimation of population size was unheard of. Meanwhile, most estimates of national prevalence were made by a handful of people working for international organizations, using assumptions that did not reflect the diversity of national epidemics [49].

Now, the picture is very different. Most countries have developed surveillance systems based around the specific risks that drive their national epidemic and gather data related to risk behaviors and treatment outcomes in ways that are comparable across time. Population size estimation allows for the development of prevalence estimates that are useful in informing programming and measuring risks. Many different sectors cooperate in the implementation of these systems, which are largely country-led [50].

This transition in HIV surveillance was made possible by the vast, disease-specific investments in HIV seen from the early 2000s, investments that were themselves triggered, in part, by findings in countries such as Thailand, an “early-adopter” of risk-based surveillance for HIV. While we do not imagine that similar investments will be forthcoming in the field of medicine quality, we believe that increased national investments in medicine procurement through expanded efforts to achieve universal health coverage will increase the urgency of ensuring that public money is invested in medicines that actually cure patients or prevent disease, rather than in their substandard or falsified doppelgangers. It is, thus, a good time to start building the capacities and systems that will improve the ways in which health systems measure; understand; and, ultimately, curtail the extent and distribution of substandard and falsified medicines.

#### Prioritizing Surveillance Approaches for Medicine Quality

As with infectious disease surveillance, the 2 approaches we have suggested for surveillance of medicine quality—case finding and sentinel surveillance—have different purposes. Case finding aims to pinpoint problems for an immediate response, while sentinel surveillance allows for more reliable quantification of the problem and for the monitoring of the effectiveness of interventions. It is unclear in situations where resources are constrained, which one a regulator should prioritize.

In the short term, especially, from the point of view of the medicine regulator who will be held responsible if substandard or falsified medicines are shown to harm patients, case finding will probably be the more attractive option. This is true despite the fact that case finding is more data intensive and, likely, more technically challenging to implement, because it requires greater specificity to succeed than sentinel surveillance. Case finding is likely to be the more valuable approach in settings where the regulator is well resourced and where substandard and falsified medicines are comparatively rare.

Where it is suspected that falsified and, especially, substandard medicines may be rather more prevalent, however, the calculus changes, at least, from a broader public health point of view. Here, a more robust understanding of the extent of the problem, provided by estimates based on sentinel surveillance, may be more valuable. In such settings, which may include many low- and middle-income countries, the prevalence of substandard and, to a lesser extent, falsified medicines will likely have a system-wide effect on health outcomes, as well as on public and private finances. Only after estimating prevalence can one reliably estimate impact. Robust estimates of impact are politically persuasive and may encourage policy makers to change health financing, procurement, and industrial policies in ways intended to erode the factors that incentivize the production and sale of substandard medicines and to shrink the market for falsified products. In addition, a clear understanding of the magnitude of the problem may prove a powerful argument for adequate resourcing for medicine regulators, especially, in lower-income settings.

We are currently consulting closely with the Indonesian national medicine regulator to trial risk-based case finding, as well as to plan and implement sentinel surveillance and develop national estimates using the methods suggested in this paper. We welcome challenges to our thinking and suggestions to improve the proposed methods, and we look forward to continued debate on the subject.

## Abbreviations

WHO: World Health Organization
